# miR32-5p promoted vascular smooth muscle cell calcification by upregulating TNFα in the microenvironment

**DOI:** 10.1186/s12865-019-0324-x

**Published:** 2020-01-17

**Authors:** Jingsong Cao, Ling Chen, Xiaoling Zhong, Yingying Shen, Yan Gao, Qian Chen, Xuyu Zu, Jianghua Liu

**Affiliations:** 1 Institute of Clinical Medicine, The First Affiliated Hospital of University of South China, Hengyang City, 421000 Hunan Province China; 2 Department of Metabolism and Endocrinology, The First Affiliated Hospital of University of South China, Hengyang, China

**Keywords:** VSMC calcification, miR32-5p, PIKfyve, TNFα, Microenvironment

## Abstract

**Background:**

Vascular calcification is often associated with chronic inflammation and is a risk factor for brain arterial stiffness. Our previous results showed that miR32-5p was positively correlated with vascular smooth muscle cells (VSMC) calcification, but it is unclear whether miR32-5p promoted VSMC calcification by regulating inflammatory factor production.

**Results:**

In this study, bioinformatics analysis was used to select tumour necrosis factor α (TNFα) as a candidate inflammatory factor associated with calcification. Moreover, alizarin red staining and qRT-PCR analysis revealed that TNFα produced by BV2 cells was the key promoting factor of VSMC calcification. Interestingly, the expression of TNFα was significantly increased at the mRNA and protein levels after miR32-5p mimic treatment but significantly decreased after miR32-5p antagomir treatment. To explore the mechanism of the regulation of TNFα expression by miR32-5p, bioinformatics analysis indicated that PIKfyve was a candidate target gene of miR32-5p, and luciferase assays verified that the expression of PIKfyve was significantly repressed by miR32-5p mimics. Importantly, rescue experiments showed that the expression of TNFα in BV2 cells treated with miR32-5p antagomir and the PIKfyve inhibitor YM201636 was significantly increased.

**Conclusions:**

The production of TNFα in microglia could be affected by miR32-5p targeting PIKfyve, and these results will be beneficial to reveal the mechanism of brain arterial calcification.

## Introduction

Vascular calcification is an independent risk factor for cardio-cerebrovascular diseases [1, 2], and its development has been associated with many factors, such as metabolic diseases, vascular diseases and even ageing [3]. Nevertheless, the key link of vascular calcification is a phenotypic change of vascular wall cells, especially the differentiation of vascular smooth muscle cells to osteoid cells [4].

One important characteristic of VSMC calcification is the upregulated expression of calcification-related genes and the downregulated expression of marker genes of smooth muscle cells [5, 6]. In the calcification process, microRNAs (miRs) play an important role in the regulation of post-transcriptional gene expression [7–9], mediated target mRNA degradation or translational repression [10, 11]. For example, miR34a promotes VSMC calcification by targeting sirtuin 1 [12], and miR29 contributes to VSMC calcification by mediating elastin downregulation [13]. Interestingly, once VSMC calcification has occurred, calcified VSMCs can produce matrix vesicles or exosomes containing miRs that affect normal cells [14]. Therefore, some researchers have proposed that miRs could be important markers in peripheral blood to predict VSMC calcification [15].

Vascular calcification, similar to other serious diseases, is involved in complicated network mechanisms, and its development is associated with not only changes in miR expression but also the induction of inflammation in the microenvironment [16, 17]. However, the mechanism by which miR affects VSMC calcification by regulating the production of inflammatory factors is still unclear.

Previous research has shown that miR32-5p promotes mouse VSMC calcification by targeting the 3′-untranslated region of phosphatase and tensin homologue mRNA [18]. In this study, miR transfection, bioinformatics analysis, dot-ELISA, qRT-PCR, luciferase assays and alizarin red staining were used to analyse the influence of miR32-5p on VSMC calcification via the regulation of inflammatory factor production in BV2 cells. The results will be useful to reveal the mechanism of brain arterial calcification.

## Materials and methods

### Cell culture

The mouse microglia cells BV2 and 293 T cells were purchased from National Infrastructure of Cell Line Resource (China Center for Type Culture Collection), and mouse VSMCs were purchased from Guang Zhou Jennio Biotech Co., Ltd. The cells were cultured in Dulbecco’s modified Eagle’s medium (DMEM, Gibco BRL, Grand Island, USA) with 10% foetal calf serum (FBS, Gibco, Australia) and 100 U/ml penicillin-streptomycin at 37 °C and 5% CO_2_.

### Bioinformatics analysis

The proteins interacting with osteoprotegerin (OPG) were analysed by Cytoscape software. Analysis of the target genes of miR32-5p was performed by using the miRDB, TargetScanVert, and TargetMiner databases and the Gene database of National Center for Biotechnology Information (NCBI). The primers were designed using primer 5 software. The restriction enzyme cutting sites were analysed by DNAMAN software.

### miR transfection and YM201636 treatment

After digestion with trypsin (Beyotime Institute of Biotechnology, China) and centrifugation at 1000 rpm, BV2 cells were collected and re-suspended in transfection medium. The cells were seeded into 12-well plates and cultured at 37 °C in 5% CO_2_ for 30 min. Following the addition of the premixed solution of Lipofectamine 2000 and miR32-5p mimics, miR32-5p antagomir or negative controls, BV2 cells were cultured at 37 °C and 5% CO_2_ for 6 h. Then, the transfection medium was removed, and the cells were cultured at 37 °C and 5% CO_2_ for 24 h after the medium was replaced with fresh culture medium or with fresh culture medium containing 0.5 μg/ml LPS (lipopolysaccharide, Beijing Solarbio Science & Technology Co., Ltd., China).

BV2 cells transfected with miR32-5p antagomir were treated with DMSO (dimethyl sulfoxide) or 10 nM YM201636 (MedChemExpress Co., Ltd., USA), which is a specific inhibitor of PIKfyve (phosphoinositide kinase, FYVE-type zinc finger containing), for 1.5 h, and then the culture medium was replaced with fresh culture medium containing 0.5 μg/ml LPS for 24 h.

### Luciferase assay

The wild-type and mutant sequences of the 3′ untranslated region (UTR) of PIKfyve were chemically synthesized, cloned into the pGL3-basic luciferase reporter plasmid (BGI, China) and named PIKfyve-3′ UTR Wt (wild type) and PIKfyve-3′ UTR Mut (mutation type). Then, 500 ng PIKfyve-3′ UTR Wt or PIKfyve-3′ UTR Mut and 50 nM miR32 mimics, 100 nM miR-32 antagomir or suitable negative controls (RiboBio Co. Ltd., China) were mixed with Lipofectamine 2000 and then transfected into 293 T cells. The cells were cultured at 37 °C, 5% CO_2_ for 48 h. After the cells were harvested, a luciferase assay was carried out with the Dual-Luciferase Reporter Assay System Kit (Promega, San Luis Obispo, CA, USA) at Turner BioSystems (Sunnyvale, CA, USA).

### Collection of BV2 cell protein and culture supernatant

The culture supernatant of BV2 cells was collected after centrifugation at 3000 rpm for 5 min. Following trypsinization and centrifugation at 1000 rpm for 5 min, BV2 cell protein was extracted with RIPA buffer (Beijing Solarbio Science & Technology Co., Ltd., China). The protein concentration of all samples was detected with a BCA protein concentration detection kit (BOSTER Biological Technology Co. Ltd., China).

### Dot-ELISA

A 1 μl sample was dropped into a nitrocellulose (NC) membrane, which had been soaked in PBS (Beijing Solarbio Science & Technology Co., Ltd., China) for 10 min and dried at room temperature (RT). After the sample was absorbed, the NC membrane was blocked in blocking buffer (PBS containing 5% nonfat dried milk (BOSTER Biological Technology Co. Ltd., China) and 0.1% Tween-20) at RT for 1 h and then washed with PBS at RT for 5 min 3 times. Subsequently, the NC membrane was incubated with rabbit anti-mouse TNFα (1:300, BOSTER Biological Technology Co. Ltd., China) or rabbit anti-mouse PIKfyve (1:300 Proteintech Group, Inc., USA), which was diluted with blocking buffer at RT for 1 h, and washed with PBS at RT for 5 min 3 times. Following incubation with goat anti-rabbit IgG-HRP (1:2000, BOSTER Biological Technology Co. Ltd., China) diluted with blocking buffer at RT for 40 min, the NC membrane was washed with PBS at RT for 15 min 2 times. Finally, the protein in the NC membrane was detected by using a Superstar ECL Plus Ready-to-use kit (BOSTER Biological Technology Co. Ltd., China) and a FluorChem E system (Cell Biosciences, USA).

### Induction of VSMC calcification

According to the report by Ma et al [19] with some modification, the VSMCs were divided into 6 groups, cultured in 12-well plates with 1 ml DMEM with 10% FBS and 100 U/ml penicillin-streptomycin, and treated as follows: group 1 received 10% BV2 culture supernatant (treated with LPS) and 10 mM β-glycerophosphate (Sigma, Poole, Dorset, UK); group 2 received 10% BV2 culture supernatant (treated with LPS), which was incubated with 7 μl rabbit anti-mouse TNFα antibody (BOSTER Biological Technology Co. Ltd., China) at 4 °C for 12 h and 10 mM β-glycerophosphate; group 3 received 10 mM β-glycerophosphate and 10 ng/ml mouse TNFα (BOSTER Biological Technology Co. ltd., China); group 4 received 10 ng/ml mouse TNFα; group 5 received 10 mM β-glycerophosphate; and group 6 received equal volume of PBS. The VSMCs of all groups were cultured at 37 °C and 5% CO_2_ for 5 days, and the culture supernatants were replaced at day 3.

### RNA extraction and cDNA synthesis

The BV2 cells in Materials and methods section 2.3 and VSMCs in Materials and methods section 2.7 were respectively collected, total RNA was extracted by RNA simple Total RNA Kit (TianGen Biotech (Beijing) Co., Ltd., China), and cDNA was synthesized by Revert Aid First Strand cDNA Synthesis Kit (Thermo Fisher Scientific Inc., Waltham, MA USA).

### qRT-PCR

The 20 μl reaction volume of qRT-PCR contained 10 μl 2 × SYBR Green PCR Mastermix (Beijing Solarbio Science & Technology Co., Ltd., China), 1 μl forward primer, and 1 μl reverse primer (Table 1), 0.2 μl ROX II (Beijing Solarbio Science & Technology Co., Ltd., China), 1 μl cDNA template and 6.8 μl ddH_2_O. The reaction programme was as follows: 95 °C for 5 min; 40 cycles at 95 °C for 15 s, 58 °C for 20 s, and 72 °C for 30 s. The experiment was performed in ABI 7500.

**Table 1:** The primers sequence

Primer name	Sequence (5′ → 3′)
PIKfyve-F	CTGGACTCTGCTAATGATTTGC
PIKfyve-R	CCTCGCTCTTTGTTAAAACGAA
TNFα-F	GGCAGGTCTACTTTGGAG
TNFα-R	TGAGTTTACCCGAAAGG
Runx2-F	AGTCCCAACTTCCTGTGCT
Runx2-R	CTGCTCCGTTCTCAAAGTGG
α-SMA-F	GGCATCCACGAAACCACCTAT
α-SMA-R	ATGAGACAGACCTAGCCACCG
GAPDH-F	TGTTTCCTCGTCCCGTA
GAPDH-R	TCACCGTTTCACCTCTAAC

### Alizarin red staining analysis

Calcified VSMCs were determined by alizarin red staining. Briefly, after washing with PBS, the VSMCs were fixed with 4% paraformaldehyde at RT for 15 min. Following staining with Alizarin Red S solution (Beijing Solarbio Science &Technology Co., Ltd., China) at RT for 2 min, samples were washed with PBS and observed under a light microscope.

### Statistical analysis

All experiments were repeated at least 3 times. The data were analysed with SigmaPlot 12.0 software and GraphPad Prism 7.00 software, and the results are shown as the mean ± S.E.M. Student’s t test or one-way ANOVA with Bonferroni correction was used to assess statistical significance, and *P* < 0.05 or *P* < 0.01 were considered significant or very significant, respectively.

## Results

### Exploratory analysis for the inflammatory factor associated with VSMC calcification

**Fig. 1:**
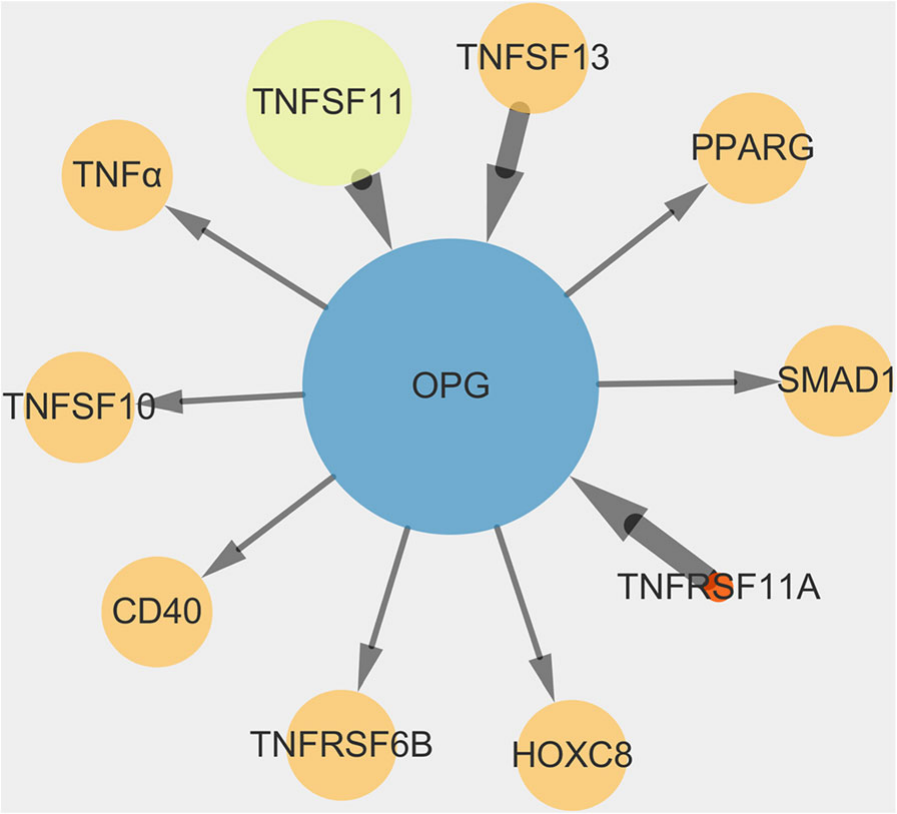
Search for inflammatory factor associated with VSMC calcification. The protein ID in the circle respectively represented as followed: TNFα, tumor necrosis factor α (P01375); TNFSF13, TNF superfamily member 13 (O75888); TNFSF11, TNF superfamily member 11 (O14788); OPG, TNF receptor superfamily member 11b (O00300); TNFRSF11A, TNF receptor superfamily member 11a (Q9Y6Q6); PPARG, peroxisome proliferator activated receptor gamma (P37231); HOXC8, homeobox C8 (P31273); SMAD1, SMAD family member 1 (Q15797); TNFSF10, TNF superfamily member 10 (P50591); TNFRSF6B, TNF receptor superfamily member 6b (O95407); CD40, CD40 molecule (P25942)

Our previous studies verified that OPG was negatively correlated with VSMC calcification. Thus, Cytoscape software was used to identify OPG interaction proteins. The results showed that the inflammatory factor TNFα (P01375) was a potential regulator involved in VSMC calcification (Fig. 1).

### The effect of TNFα expressed by BV2 cells on VSMC calcification

**Fig. 2:**
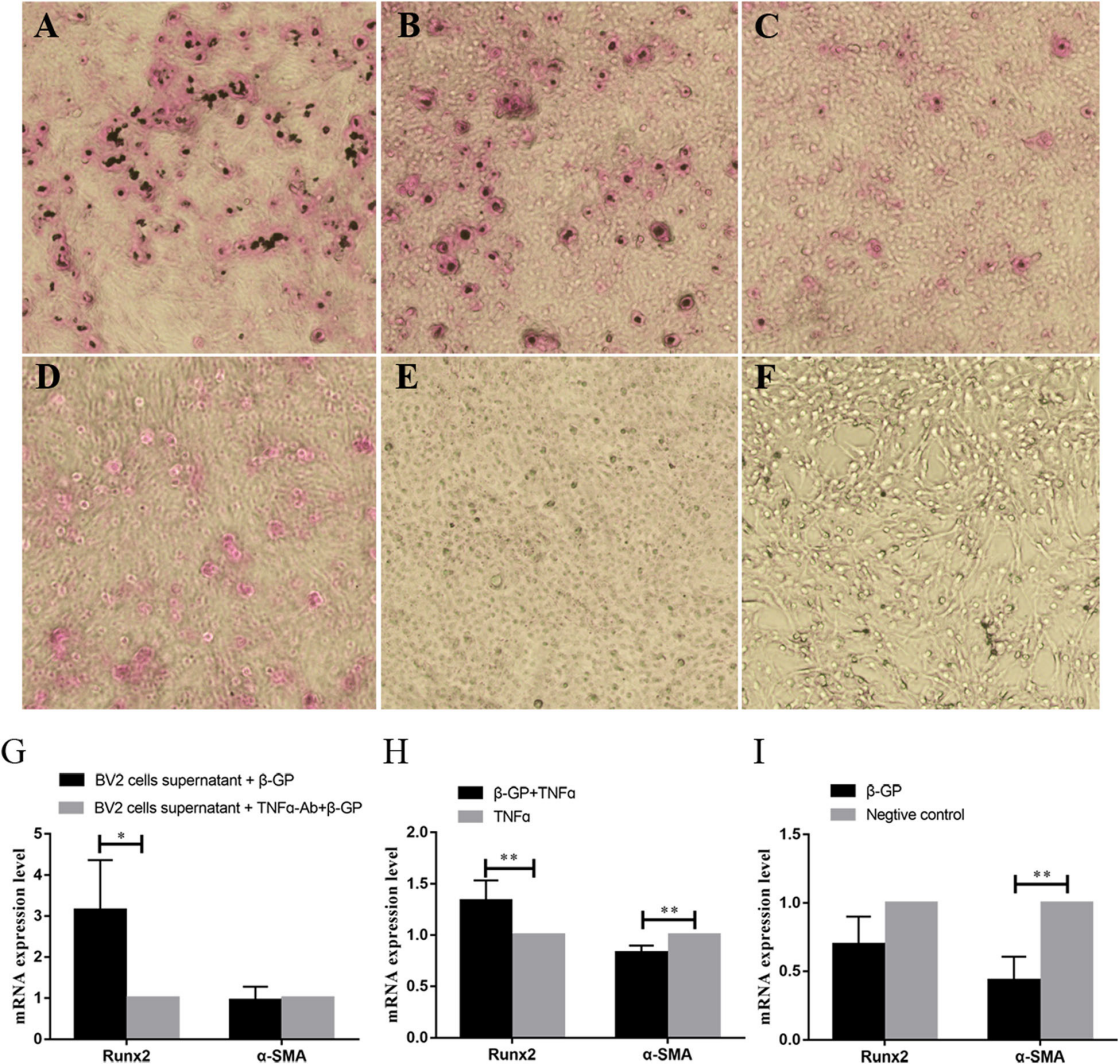
The influence of TNFα expressed by BV2 cells for VSMC calcification. **a**-**f** Alizarin red staining analysis of VSMC calcification in differently stimulated condition (40×), **a** group1 added 10% BV2 culture supernatant and 10 mM β-glycerophosphate, **b** group3 added 10 mM β-glycerophosphate and 10 ng/ml mouse TNFα, **c** group5 10 mM β-glycerophosphate, **d** group2 added 10% BV2 culture supernatant, which was incubated with rabbit anti-mouse TNFα antibody, and 10 mM β-glycerophosphate, **e** group4 added 10 ng/ml mouse TNFα, **f** group6 added equal volume PBS. **g-i** qRT-PCR analysis for the expression of Runx2 and α-SMA in VSMC, **g** the mRNA expression of Runx2 and α-SMA in groups 1 and 2, **h** the mRNA expression of Runx2 and α-SMA in groups 3 and 4, **i** the mRNA expression of Runx2 and α-SMA in groups 5 and 6. Bars represent mean ± S.D. (*n* ≥ 3). Significant difference marked with one (*p* < 0.05) or two (*p* < 0.01) asterisks

Furthermore, the effect of TNFα on VSMC calcification was verified (Fig. 2). The results showed that the calcification of VSMCs was promoted by BV2 cell culture supernatant and TNFα after β-glycerophosphate induction (Fig. 2a, b), whereas BV2 culture supernatant incubated with TNFα antibody (Fig. 2d) or TNFα by itself (Fig. 2e) did not. In addition, qRT-PCR showed that the expression of Runx2 (RUNX family transcription factor 2) significantly increased 2.80- and 1.34-fold in group 1 and 3, respectively, and increased 1.08-fold in group 5; the expression of α-SMA (α-Smooth muscle actin) decreased 0.95-fold in group 1 and significantly decreased 0.83- and 0.43-fold in group 3 and group 5, respectively (Fig. 2g-i). These results suggested that among the inflammatory factors produced by BV2 cells, TNFα significantly promoted VSMC calcification.

### The effect of miR32-5p on TNFα expression in BV2 cells

**Fig. 3:**
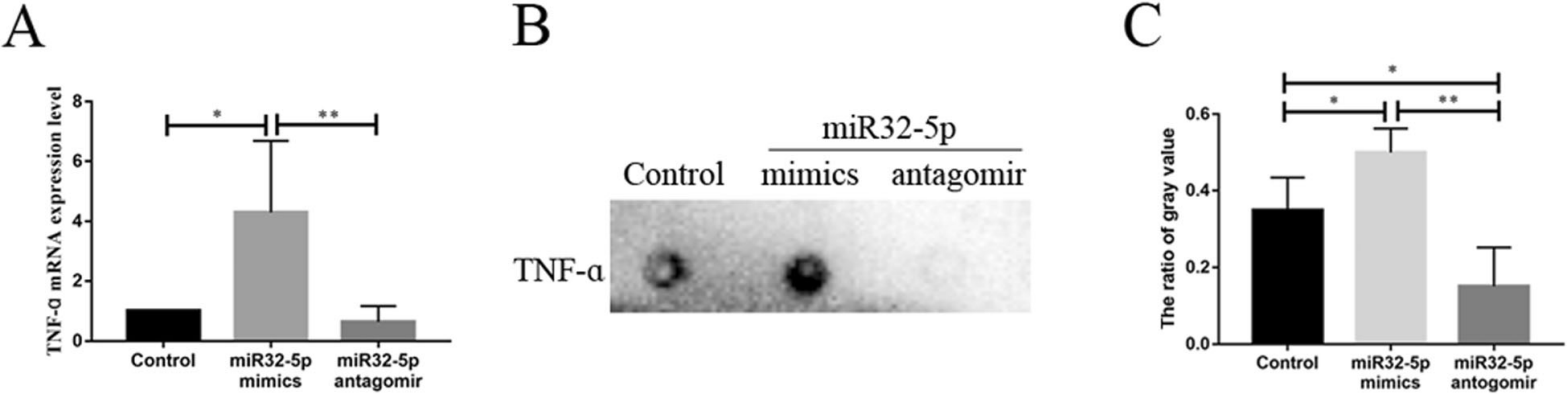
miR32-5p up-regulated TNFɑ producing in BV2 cells. **a** The mRNA expression of TNFɑ in BV2 cells after LPS stimulated by qRT-PCR analyzed. **b** The protein expression of TNFɑ in BV2 cells after LPS stimulated by Dot-ELISA analyzed. **c** The gray value analysis of Dot-ELISA. Bars represent mean ± S.D. (*n* ≥ 3). Significant difference marked with one (*p* < 0.05) or two (*p* < 0.01) asterisks

To reveal the roles of miR32-5p in the expression of TNFα, BV2 cells were transfected with miR32-5p mimics or miR32-5p antagomir. The results showed that TNFα mRNA was significantly increased 4.30-fold after transfection with mimics but significantly decreased 0.63-fold after antagomir treatment (Fig. 3a). Moreover, a dot-ELISA assay showed that TNFα in BV2 cells treated with mimics and antagomir significantly increased 1.43-fold and decreased 0.43-fold, respectively (Fig. 3b, c). The results suggested that miR32-5p promoted the expression of TNFα in BV2 cells.

### Identification of the target gene of miR32-5p

**Fig. 4:**
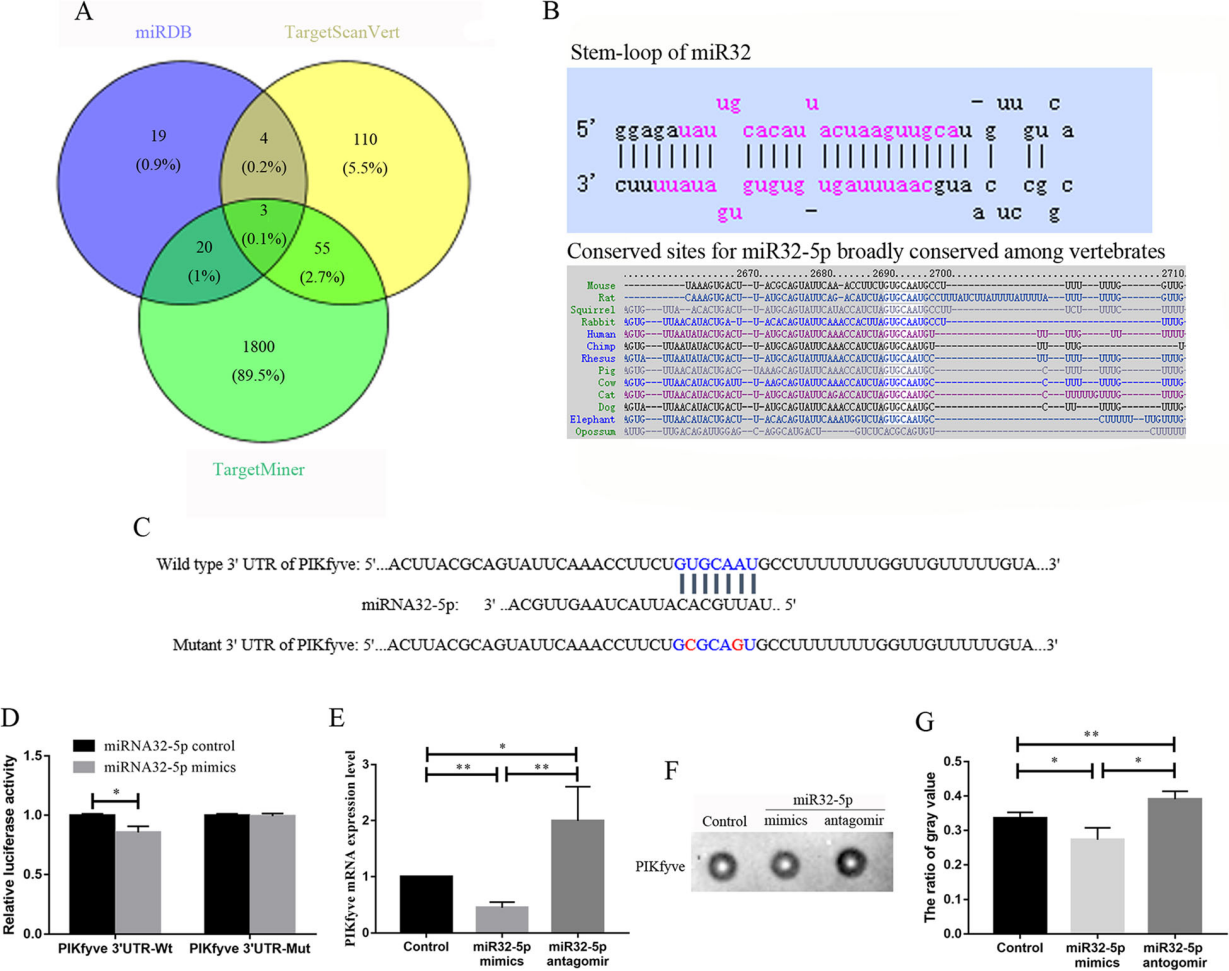
Analysis of the target gene of miR32-5p. **a** The candidate target genes of miR32-5p by bioinformatics analyzed, the three genes in intersection were RSBN1, PIKfyve and ZFC3H1. **b** The stem-loop of miR32 and the conserved sites in the 3′ UTR of PIKfyve for miR32-5p. **c** The wild type and mutation PIKfyve3’ UTR of the seed region of miR-32. **d** The analysis of miR32-5p target PIKfyve by luciferase assays. **e** The expression of PIKfyve in BV2 cells after transfected with miR32-5p mimics or antagomir by qRT-PCR analysis. **f** The expression of PIKfyve in BV2 cells after transfected with miR32-5p mimics or antagomir by Dot-ELISA analysis. **g** The gray value analysis for Dot-ELISA. Bars represent mean ± S.D. (n ≥ 3). Significant difference marked with one (*p* < 0.05) or two (*p* < 0.01) asterisks

To reveal the mechanism of TNFα expression regulated by miR32-5p, the potential target genes of miR32-5p were searched and analysed by the miRDB, TargetScanVert, TargetMiner and NCBI databases, and PIKfyve was selected as a candidate target gene of miR32-5p (Fig. 4a). Based on the search results in the miRDB database, the sequence of the miR32-5p binding site in PIKfyve (wild type and mutant type) was designed (Fig. 4b, c). Furthermore, luciferase assays verified that the relative luciferase activity in 293 T cells was significantly downregulated 0.85-fold after cotransfection with PIKfyve 3’UTR-Wt and miR32-5p mimics, while there was no significant change after treatment with PIKfyve 3’UTR-Mut and miR32-5p mimics (Fig. 4d).

In addition, qRT-PCR assay showed that compared with that in the control group, PIKfyve in BV2 cells transfected with miR32-5p mimics or antagomir significantly increased 0.45-fold or decreased 2.00-fold, respectively (Fig. 4f). Dot-ELISA determined that PIKfyve in BV2 cells transfected with miR32-5p mimics was significantly downregulated 0.81-fold and significantly upregulated 1.16-fold in BV2 cells transfected with miR32-5p antagomir (Fig. 4e, g).

### PIKfyve inhibited TNFα expression in BV2 cells

**Fig. 5:**
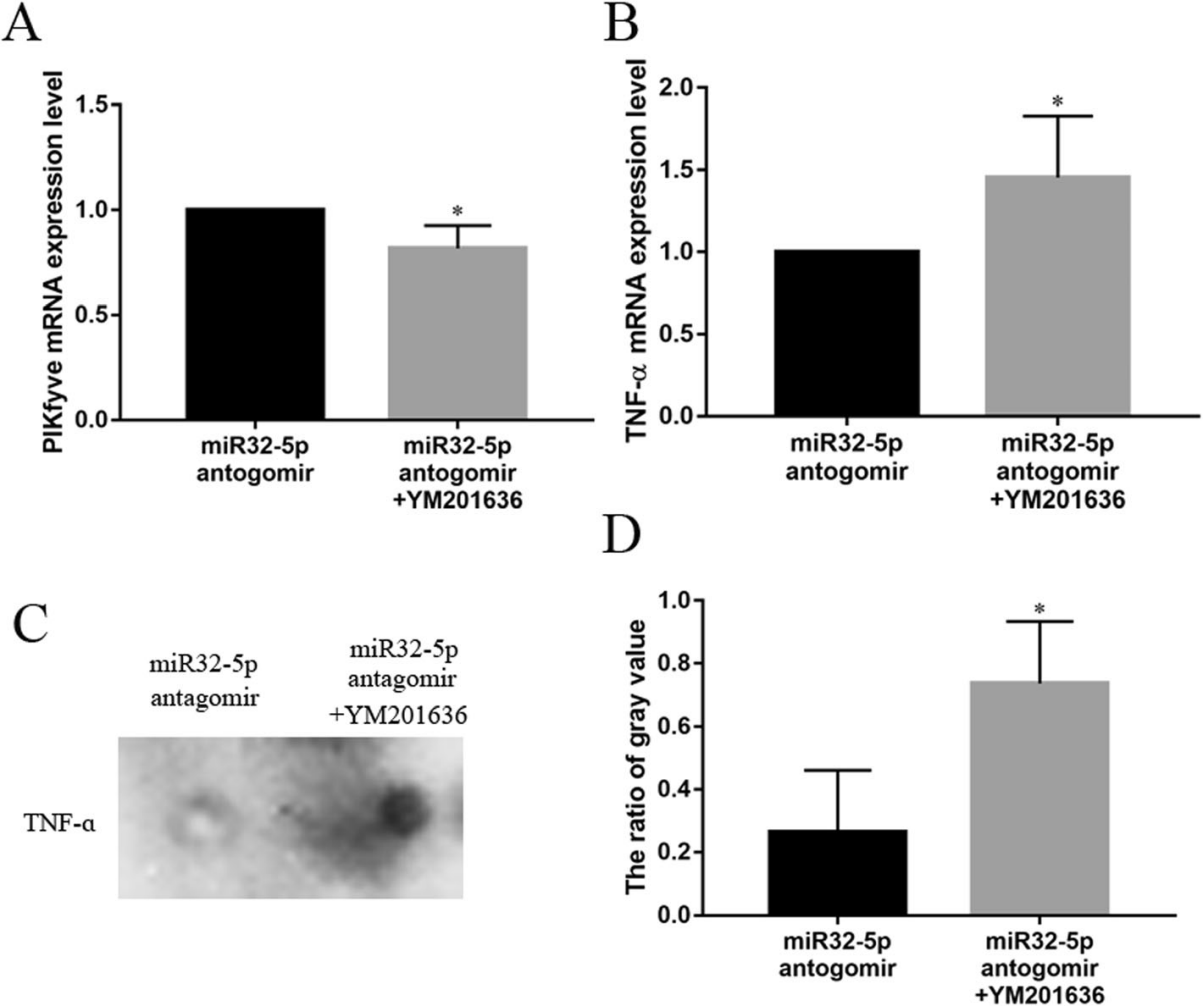
The influence of PIKfyve for TNFɑ production in BV2 cells by qRT-PCR and Dot-ELISA analysis. **a** The expression changes of PIKfyve after treated with YM201636 by qRT-PCR analysis. **b** The expression changes of TNFɑ after treated with YM201636 by qRT-PCR analysis. **c** The expression changes of TNFɑ after treated with YM201636 by Dot-ELISA analysis. **d** The gray value analysis for Dot-ELISA. Bars represent mean ± S.D. (n ≥ 3). Significant difference marked with one (*p* < 0.05) or two (*p* < 0.01) asterisks

To investigate the effect of PIKfyve on TNFα expression, BV2 cells transfected with miR32-5p antagomir were treated with DMSO or YM201636. After YM201636 treatment, PIKfyve mRNA was significantly decreased 0.82-fold (Fig. 5a). Moreover, the TNFα mRNA and protein levels increased significantly 1.45- and 4.43-fold, respectively (Fig. 5b-d). The results suggested that PIKfyve inhibited TNFα production.

## Discussion

Vascular calcification is a complex pathological process. Its development is often involved in diseases with chronic inflammatory disorders [20–22], such as type 2 diabetes mellitus and brain arterial stiffness [2, 23]. In the central nervous system, microglia, which are the largest population of mononuclear phagocytes [24], play important roles in perivascular and pericalcification areas [25]. Interestingly, microglia activation can be induced by high levels of glucose [26], and activated microglia are extensively perivascularly distributed [27].

So, based on previous results that OPG was an inhibitor in VSMC calcification while miR32-5p was a promoter [18], OPG, also named TNF receptor superfamily member 11b was selected to explore the inflammatory factors which was associated to VSMC calcification (Fig. 1). Among the candidate proteins, TNFα can promote the development of vascular calcification through interactions with TNF receptor 1 [28–30].

Subsequently, in cytokines produced by microglia, the importance of TNFα for VSMC calcification was analysed. The results showed that the calcification rate of VSMCs was promoted by TNFα (Fig. 2). Moreover, qRT-PCR verified that Runx2 and α-SMA were up- and downregulated, respectively, in obviously calcified VSMCs (Fig. 2). Interestingly, VSMC calcification was not obviously affected by TNFα on its own, unless β-glycerophosphate was added. Thus, TNFα was a key inducer in BV2 cell culture supernatants.

Furthermore, considering higher miR32 levels in plasma of patients with coronary artery calcification, the influence of miR32-5p for microglia producing TNFα was analysed (Fig. 3). The results showed that TNFα increased expression after miR32-5p mimics treated while decreased expression after miR32-5p antagomir treated. Though the results appeared the regulation of miR32-5p for TNFα, these data disagreed with the role of miRNAs, which is degradation or translational repression of the target mRNA [31]. Thus, there is likely a gene that mediates the action of miR32-5p on TNF.

Therefore, the target genes of miR32-5p were explored by bioinformatics method (Fig. 4a). Among the three common genes (RSBN1, PIKfyve and ZFC3H1), PIKfyve plays important roles in the regulation of pleiotropic cellular functions [32–34], for instance regulating the function of macrophage through interfering phagosome and endosome maturation [35]. Meanwhile, luciferase assays appeared that the luciferase activity decreased significantly after transfecting with miR32-5p mimics and the recombination plasmid of PIKfyve 3’UTR-Wt (Fig. 4b-d). Furthermore, qRT-PCR and dot-ELISA showed that the expression of PIKfyve at the mRNA and protein levels was decreased by miR32-5p expression (Fig. 4f-g). These results suggested that PIKfyve was a target gene of miR32-5p.

To determine the effect of PIKfyve on TNFα production in BV2 cells, a rescue experiment was carried out. After BV2 cells were treated with the miR32-5p antagomir and the PIKfyve-specific inhibitor YM201636, the PIKfyve mRNA was downregulated, while TNFα mRNA was upregulated (Fig. 5a, b). In addition, the production of TNFα in the BV2 cell culture supernatant was increased (Fig. 5c, d). This result was similar to the study by Kawasaki et al., which reported that TNFα production in mouse embryonic fibroblast cells was negatively correlated with the expression of PIKfyve [36]. These results suggested that PIKfyve inhibited the producing of TNFα in BV2 cells.

## Conclusions

Overall, this study found that miR32-5p/PIKfyve/TNFα is a signaling axis that regulates TNFα production in BV2 cells, while TNFα could significantly promote VSMC calcification. The results will be valuable to reveal the mechanism of brain arterial calcification.

## Data Availability

All data in the article can be requested from the corresponding author.

## Abbreviations

DMEM: Dulbecco’s modified Eagle’s medium
DMSO: Dimethyl sulfoxide
FBS: Foetal calf serum
LPS: Lipopolysaccharide
α-SMA: α-Smooth muscle actin
miR: microRNA
Mut: Mutation type
NC: Nitrocellulose
NCBI: The Gene database of National Center for Biotechnology Information
OPG: Osteoprotegerin
PIKfyve: Phosphoinositide kinase, FYVE-type zinc finger containing
RT: Room temperature
Runx2: RUNX family transcription factor 2
TNFα: Tumour necrosis factor α
UTR: Untranslated region
VSMC: Vascular smooth muscle cells
Wt: Wild type
